# Adherence to the USDA dietary recommendations for fruit and vegetable intake and risk of fatal stroke among ethnic groups: a prospective cohort study

**DOI:** 10.1186/1471-2377-13-120

**Published:** 2013-09-13

**Authors:** Sangita Sharma, Mohammadreza Pakserescht, Kennedy Cruickshank, Deborah M Green, Laurence N Kolonel

**Affiliations:** 1 Department of Medicine, University of Alberta, 5-10 University Terrace, 8303 112 Street, Edmonton, Alberta T6G 2T4, Canada; 2 Cardiovascular Sciences, University of Manchester, 3rd floor CTF, 46 Grafton St, Manchester M13 9NT, UK; 3 Departments of Neurology and Neurosurgery, Neurocritical Care, Boston University School of Medicine, Collamore Building C3, 72 East Concord Street, Boston, MA, USA; 4 Cancer Research Center of Hawaii, University of Hawaii, 1236 Lauhala Street, Honolulu, Hawaii 96813, USA

**Keywords:** Fruits, Vegetables, Stroke mortality, Ethnicity

## Abstract

**Background:**

Stroke is the fourth leading cause of death in the U.S. and stroke mortality rates differ substantially by ethnic group. The impact of adherence to the USDA dietary guidelines on risk for fatal stroke among different ethnic groups has not previously been examined.

**Methods:**

A prospective cohort design was used to examine associations between adherence with dietary recommendations for fruit and vegetable intake and risk for stroke mortality among 174,888 men and women representing five ethnic groups; African American, Native Hawaiian, Japanese American, Latino, and Caucasian. Dietary intake was assessed using a mailed quantitative food frequency questionnaire. Associations were examined using Cox proportional hazards models.

**Results:**

There was no evidence that ethnicity modified associations between fruit and vegetable intake and stroke mortality. When data for different ethnicities were combined, a reduced risk for fatal stroke was observed among women who were adherent with the USDA dietary recommendations for vegetable intake, although this result did not reach statistical significance (RR = 0.84, 95% CI = 0.68-1.04). No associations were observed among men.

**Conclusions:**

The results of this study do not provide evidence that dietary intake of fruits and vegetables differentially impacts risk for stroke mortality among different ethnic groups.

## Background

Stroke (cerebrovascular disease) was the fourth leading cause of death in the United States in 2008, and accounted for 134,148 deaths that year [1]. Mortality from stroke differs substantially by ethnic group within the U.S. In 2008, the age-adjusted death rates from stroke among men (per 100,000 population), were 63.4 for African Americans, 39.2 for Caucasians, 24.5 for American Indians, 33.1 for Latinos, and 34.0 for Asians. The ethnic-specific rates among women were similar to those for men for Caucasians, American Indians, and Asians, but were considerably lower among the African American and Hispanic women (54.5 and 28.9, respectively) [1]. The rates of stroke fatalities are also increasing in developing countries, indicating that modifiable lifestyle factors, including poor diet, might be the main contributors to risk rather than genetic differences [2,3].

Diet is a known modifiable risk factor for stroke, and its main risk factor, hypertension [4]. The protective effect of fruit and vegetables on risk of stroke has been reported in large prospective population-based studies [5-7]. Previous studies on the Multiethnic Cohort (MEC) have shown that adherence with dietary recommendations and food group consumption differs substantially by ethnic group [8,9]. In addition, ethnic variations in risk factors have also been reported in the MEC study [10]. The prevalence of hypertension among MEC participants varied by ethnicity, ranging from 29.2%, 35.2%, 40.0%, 44.6%, to 55.5%, among Caucasians, Latinos, Japanese, Native Hawaiians and African Americans, respectively [10]. The proportion of the U.S. population from minority ethnic backgrounds is increasing and there is a recognized need for additional health data on how risk factors and rates of disease vary by ethnicity within the U.S. population [11,12]. The United States Department of Agriculture (USDA) has established dietary guidelines for daily consumption of fruits and vegetables [13]. Although a few studies have compared adherence to the USDA dietary recommendations using the healthy eating index with risk of chronic disease [14-16], none have examined adherence to the USDA dietary recommendations by ethnic group.

The objective of the present study was to examine associations between adherence to the USDA dietary recommendations for fruit and vegetable intake and risk for fatal stroke, and to determine if this effect varied by ethnicity.

## Methods

### Study population

The Multiethnic Cohort (MEC) included large representative samples of men and women aged 45 to 75 years from five ethnic groups; Caucasian, African American, Native Hawaiian, Japanese American, and Latino. Details of the study design and recruitment are reported elsewhere [17]. Briefly, the target study population was identified using drivers’ license files supplemented with other sources for persons living in the state of Hawaii and the Los Angeles County area of the United States between 1993 and 1996. Potential participants were mailed a comprehensive self-report questionnaire including a detailed quantitative food frequency questionnaire (QFFQ), in addition to questions on demography, anthropometry, medication use, history of medical conditions (including self-reported stroke and hypertension), and other lifestyle factors [17].

A total of 201,257 participants from the target ethnic groups responded to the questionnaire, with the response rate ranging from 20% in Latinos to 49% in Japanese-Americans. For this analysis, those with missing smoking information (n = 6,743), implausible diets based on energy and macronutrient intakes as well as daily food group servings (n = 12,346), or with implausible or missing anthropometric information (2,803), and subjects with a history of stroke (n = 4,609) were excluded, leaving 78,844 men and 96,044 women for the present analyses. The University of Hawaii and University of Southern California Institutional Review Boards approved the study protocol and all participants provided informed consent.

### Dietary assessment

The QFFQ was developed from three-day measured dietary records maintained by a sample of 300 subjects representative of all ethnic-sex groups to select food items which contributed at least 85% to the consumption of nutrients of interest [17]. These foods were complemented by ethnic-specific dishes, irrespective of their nutrient contribution. The QFFQ included 8–9 frequency categories ranging from ‘Never or hardly ever’ to ‘2 or more times a day’ in addition to 3–4 portion sizes specific for each food item, which were also presented in photographs.

A validation and calibration substudy using three repeated 24-hour dietary recalls collected from 260 subjects in each sex-ethnic group showed that the average correlation coefficients for all nutrients ranged from 0.26 in African American women to 0.57 in Caucasian men [18]. For nutrient densities, average correlations were about twice as high, with a range from 0.57 to 0.74 across ethnic-sex strata. Fruits and vegetables contributed to 70-75% of vitamin C intake among the different ethnic-sex groups; vegetables were major contributors to vitamin A intake (carrots alone contributed 17-35% among the different ethnic-sex groups) [Sharma, unpublished data].

Nutrients were determined using a customized and ethnic-specific food composition table (FCT) which was based on the USDA nutrient database and additional laboratory analyses performed in Hawaii [17]. The FCT included a large recipe database as well as unique ethnic-specific foods consumed by this multiethnic population. The food groups examined in this study were based on the U.S. dietary guidelines for vegetables (dark green, deep yellow, potato, starchy, tomato, other vegetables) and fruit (citrus, melons and berries, other fruits – from all sources, including juices and processed fruits) [13]. An individual’s serving of each food group was computed by summing all servings across the appropriate food items on the QFFQ. The USDA food pyramid recommendations for fruit and vegetable consumption are based on caloric intake. For caloric intakes of<1,600 kcal, 1,601-2,200 kcal, 2,201-2,800 kcal, and>2,800 kcal per day, the food guide recommendations are 3, 3, 4, and 5 servings per day for vegetables, and 2, 3, 3, and 4 servings per day for fruit. Adherence was categorized as a binary variable, determined from the percentage (<100% versus ≥100%) of the daily servings of fruits and vegetables over the recommended servings at the matched age and caloric intake level. Adherence with the dietary recommendations varied by sex and ethnicity, ranging from 36-54% among men and 48-66% among women for fruit intake, and from 43-62% among men and from 51-69% among women for vegetable intake. Further details on adherence levels and average food group intake among the MEC participants have been previously reported [8,9].

### Identification of stroke deaths

The International Classification of Disease (ICD) - 9 codes 430–438, and ICD-10 codes I60 - I64, I67 and I69 were used to identify stroke deaths among the MEC participants using state death files and the U.S. National Death Index.

### Statistical methods

Cox proportional hazards models using age as the time metric were used to calculate relative risk (RR) and 95% confidence intervals (95% CI) for risk of fatal stroke and associations with adherence to USDA dietary guidelines for fruit and vegetable consumption. Person-times were determined from the date of cohort entry to the date of death, or December 31^st^ 2001, the end date for this study. Adherence to the recommendations for each food group was categorized into quintiles based on cut-points for the entire cohort and trends were determined by calculating the risk for every 1% increment in adherence to the USDA dietary recommendation. Models based on a binary exposure variable (adherent versus non-adherent with dietary recommendations) were also examined. Stratified models were used to examine variations in the outcome measure by sex and ethnicity [19]. Models were adjusted for the following covariates: ethnicity (as strata variables), time on study (≤2, 3–5, and>5 years, as strata variables), maximum years of education, energy intake (logarithmically transformed), smoking, body mass index, physical activity (defined as average hours of moderate or vigorous physical activity per day), history of diabetes, and alcohol intake. Dietary intake of other food groups (dairy, grains, meat/meat alternatives) which have been associated with risk for stroke [20,21] were also included as covariates. A high percentage of stroke cases reported hypertension, and this factor was also highly correlated with diabetes, thus this factor was not included as a covariate. History of hormone replacement therapy was included as a covariate in the models among women. The proportional hazards assumption, as tested by examining Schoenfeld residuals, was not violated.

Due to the small number of Native Hawaiian fatal stroke cases (18 men and 26 women), ethnic-specific results could not be determined for this group. All statistical analyses were performed using SAS statistical software, version 9.1 (SAS Institute, Inc., Cary, NC).

## Results

After a follow-up of eight years, 434 male and 426 female fatal stroke cases were identified. The mean daily intakes of energy and percentages of energy from total fat, saturated fat, and alcohol, and mean daily serving intakes of vegetables, fruit, meat, dairy, and grain products were similar among cases and the entire cohort (Table 1). Fatal stroke cases had a higher prevalence of diabetes and hypertension, higher mean pack-years of cigarette smoking compared to all participants, and a higher percentage of individuals with 10 or less years of education. Cases were also less likely to be married, particularly among women.

**Table 1:** Characteristics of the total participants and cases of death from stroke

	**Men**	**Women**		
	**Cases**	**Total participants**	**Cases**	**Total participants**
	**(n = 434)**	**(n = 78,844)**	**(n = 426)**	**(n = 96,044)**
**Age at cohort entry (years)**	66.6 ± 7.1	65.7 ± 7.6	66.4 ± 7.4	59.3 ± 8.8
**Energy intake (kcal)**	2,141 ±1,009	2,301 ± 974	1,806 ± 850	1,887 ± 840
% energy from fat	30.1 ± 7.5	30.2 ± 7.1	30.2 ± 7.2	29.7 ± 7.1
% energy from saturated fat	8.9 ± 2.8	8.9 ± 2.7	9.0 ± 2.6	8.7 ± 2.6
% energy from alcohol	3.8 ± 8.2	4.1 ± 7.4	1.7 ± 5.4	1.6 ± 4.6
% energy from carbohydrate	51.3 ± 9.9	51.1 ± 9.1	52.9 ± 9.5	53.6 ± 8.9
**Food group intake (servings/day)**				
Vegetables	4.3 ± 2.7	4.5 ± 2.8	4.2 ± 2.6	4.6 ± 2.9
Fruit	3.2 ± 2.5	3.0 ± 2.6	3.4 ± 2.6	3.5 ± 2.9
Meat	5.7 ± 3.6	6.2 ± 3.8	4.8 ± 3.1	4.8 ± 3.1
Dairy	1.2 ± 1.0	1.2 ± 1.0	1.2 ± 1.0	1.2 ± 1.0
Grain	7.8 ± 3.9	8.4 ± 3.8	6.4 ± 3.3	7.0 ± 3.5
**Hours in moderate or vigorous activity per day**	1.1 ± 1.4	1.3 ±1.5	0.9 ± 1.1	1.1 ± 1.2
**Pack-years (number of cigarettes per day x years smoked / 20)**	17.4 ± 17.9	13.7 ± 16.4	10.5 ± 15.5	6.5 ± 12.0
**Ethnicity (%)**				
Caucasian	17	26	21	25
African American	25	13	36	19
Native Hawaiian	4	7	6	7
Japanese American	31	31	18	28
Latino	23	23	19	21
**Education**				
Graduated college	18	30	15	25
Grade 11/12 - some college	57	54	60	58
≤10 yrs education	25	16	25	17
**Currently Married (%)**	74	77	47	59
**Body Mass Index (kg/m**^**2**^**) (%)**				
≤ 18.5	1	1	5	3
18.6 – 25.0	49	42	41	47
25.1 – 30.0	34	43	30	31
> 30.0	16	14	24	19
**Smoking status (%)**				
Never smoker	27	31	45	57
Past smoker	51	51	33	29
Current smoker	22	18	22	14
**Repeatedly consumed alcohol (%)**	51	63	30	39
**Medical history (%)**				
History of diabetes	26	12	24	10
History of hypertension	60	39	62	37

The multivariable model results for the associations with adherence to dietary recommendations and risk for fatal stroke among men are shown in Table 2. With the exception of a significant increase in risk among African American men with intake between 51 and 75% of the recommended vegetable intake (compared to<50%: RR = 2.14, 95% CI = 1.08-4.21), there were no other significant associations between adherence with dietary recommendations and stroke mortality among men. Among women (Table 3), those of Japanese descent who adhered to the recommended daily servings for fruit consumption had a significant reduction in the risk for stroke mortality (adherent versus non-adherent: RR = 0.55, 95% CI = 0.34-0.91), and the results based on the categorical variable also suggested a decreasing risk for stroke fatality with increasing fruit consumption among this group. Adherence to the USDA recommended vegetable intake level also appeared to reduce risk of death from stroke in African American and Caucasian women, although these associations did not reach statistical significance (adherent versus non-adherent: RR = 0.70, 95% CI = 0.49-1.01 and RR = 0.66, 95% CI = 0.42-1.04; for African American and Caucasian women, respectively).

**Table 2:** Relative risk (RR)^* ^for stroke mortality associated with adherence to the USDA dietary recommendations for fruit and vegetable intake among men**

	**African American**	**Japanese American**	**Latino**	**Caucasian**	All ethnic groups***
**Cases / Non-cases**	106 / 10,323	136 / 23,978	100 / 18,177	74 / 20,615	434 / 78,410
	**RR (95**% **CI)**	**RR (95**% **CI)**	**RR (95**% **CI)**	**RR (95**% **CI)**	**RR (95**% **CI)**
**Vegetables**					
Q1 (0-50%)	1	1	1	1	1
Q2 (51-75%)	**2.14 (1.08-4.21)**	0.89 (0.35-2.25)	0.92 (0.45-1.89)	1.13 (0.48-2.65)	1.26 (0.87-1.84)
Q3 (76-100%)	1.04 (0.46-2.34)	1.32 (0.57-3.06)	1.00 (0.49-2.04)	0.91 (0.39-2.16)	1.05 (0.71-1.55)
Q4 (101-150%)	1.90 (0.91-3.93)	1.22 (0.54-2.79)	0.51 (0.24-1.10)	0.85 (0.37-1.96)	0.99 (0.68-1.44)
Q5 (≥151%)	1.67 (0.73-3.81)	1.50 (0.65-3.45)	0.97 (0.46-2.04)	0.95 (0.40-2.27)	1.28 (0.87-1.89)
1% increase of adherence	1.00 (0.99-1.00)	1.00 (0.99-1.00)	1.00 (0.99-1.00)	1.00 (0.99-1.00)	1.00 (0.99-1.00)
Binary Exposure: Adherent versus Non-Adherent	1.24 (0.81-1.91)	1.19 (0.82-1.73)	0.72 (0.47-1.11)	0.89 (0.55-1.44)	0.99 (0.81-1.22)
**Fruit**					
Q1 (0-50%)	1	1	1	1	1
Q2 (51-75%)	0.88 (0.48-1.60)	0.86 (0.46-1.64)	0.73 (0.35-1.52)	0.59 (0.24-1.41)	0.80 (0.57-1.12)
Q3 (76-100%)	0.99 (0.53-1.85)	1.43 (0.81-2.52)	1.23 (0.65-2.34)	1.27 (0.62-2.61)	1.22 (0.90-1.61)
Q4 (101-150%)	0.97 (0.55-1.71)	1.34 (0.78-2.28)	1.25 (0.69-2.28)	1.03 (0.52-2.06)	1.16 (0.87-1.54)
Q5 (≥151%)	0.73 (0.40-1.34)	1.40 (0.82-2.41)	0.92 (0.50-1.69)	1.29 (0.66-2.55)	1.10 (0.82-1.47)
1% increase of adherence	0.99 (0.99-1.00)	1.00 (0.99-1.00)	1.00 (0.99-1.00)	1.00 (0.99-1.00)	1.00 (0.99-1.00)
Binary Exposure: Adherent versus Non-Adherent	0.88 (0.58-1.33)	1.25 (0.87-1.79)	1.08 (0.71-1.63)	1.22 (0.76-1.96)	1.13 (0.92-1.37)

* Relative risk for adherent versus non-adherent, adjusted by Cox regression with age as the time metric for ethnicity, time on study, maximum years of education, energy intake (logarithmically transformed), smoking (including current smoking, past smoking and pack-years), dietary intake of dairy, grains and meat/meat alternatives, body mass index, physical activity (defined as average hours of moderate or vigorous physical activity per day), history of diabetes, and alcohol intake.

** Statistically significant values are indicated in bold.

*** Includes Native Hawaiians (18 and 26 cases, among men and women, respectively).

**Table 3:** Relative risk (RR)^* ^for stroke mortality associated with adherence to the USDA dietary recommendations for fruit and vegetable intake among women**

	**African American**	**Japanese American**	**Latino**	**Caucasian**	All ethnic groups***
**Cases /Non-cases**	152 / 18,082	76 / 27,137	80 / 19,473	80 / 19,473	92 / 24,114
	**RR (95**% **CI)**	**RR (95**% **CI)**	**RR (95**% **CI)**	**RR (95**% **CI)**	**RR (95**% **CI)**
**Vegetables**					
Q1 (0-50%)	1	1	1	1	1
Q2 (51-75%)	0.98 (0.54-1.76)	0.82 (0.19-3.48)	0.75 (0.26-2.17)	0.98 (0.44-2.20)	0.89 (0.60-1.34)
Q3 (76-100%)	1.17 (0.66-2.10)	1.66 (0.46-5.89)	1.33 (0.52-3.42)	1.05 (0.48-2.27)	1.18 (0.81-1.73)
Q4 (101-150%)	0.85 (0.47-1.53)	2.08 (0.61-7.14)	1.45 (0.58-3.59)	0.56 (0.25-1.27)	0.94 (0.64-1.37)
Q5 (≥151%)	0.63 (0.33-1.20)	1.25 (0.34-4.54)	1.15 (0.44-3.02)	0.79 (0.36-1.74)	0.82 (0.55-1.21)
*1*% *increase of adherence*	0.99 (0.99-1.00)	1.00 (0.99-1.002)	1.00 (0.99-1.00)	1.00 (0.99-1.00)	1.00 (0.99-1.00)
Binary Exposure: Adherent versus Non-Adherent	0.70 (0.49-1.01)	1.33 (0.77-2.29)	1.24 (0.73-2.09)	0.66 (0.42-1.04)	0.84 (0.68-1.04)
**Fruit**					
Q1 (0-50%)	1	1	1	1	1
Q2 (51-75%)	1.11 (0.63-1.94)	1.25 (0.58-2.69)	0.73 (0.27-1.99)	0.74 (0.34-1.60)	0.98 (0.69-1.40)
Q3 (76-100%)	0.72 (0.38-1.36)	0.82 (0.35-1.91)	1.23 (0.49-3.12)	0.72 (0.33-1.58)	0.89 (0.62-1.29)
Q4 (101-150%)	0.96 (0.57-1.63)	0.68 (0.32-1.46)	1.59 (0.73-3.45)	0.89 (0.46-1.70)	0.99 (0.72-1.36)
Q5 (≥151%)	0.85 (0.52-1.41)	0.48 (0.23-1.03)	1.47 (0.70-3.10)	0.83 (0.44-1.56)	0.88 (0.65-1.20)
*1*% *increase of adherence*	1.00 (0.99-1.00)	0.99 (0.99-1.00)	1.00 (0.99-1.00)	1.00 (0.99-1.00)	1.00 (0.99-1.00)
Binary Exposure: Adherent versus Non-Adherent	0.96 (0.68-1.35)	**0.55 (0.34-0.91)**	1.55 (0.93-2.59)	1.03 (0.66-1.61)	0.97 (0.75-1.19)

* Relative risk for adherent versus non-adherent, adjusted by Cox regression with age as the time metric for ethnicity, time on study, maximum years of education, energy intake (logarithmically transformed), smoking (including current smoking, past smoking and pack-years), dietary intake of dairy, grains and meat/meat alternatives, body mass index, physical activity (defined as average hours of moderate or vigorous physical activity per day), history of diabetes, and alcohol intake.

** Statistically significant values are indicated in bold.

*** Includes Native Hawaiians (18 and 26 cases, among men and women, respectively).

Although there appeared to be some variation in the point estimates for the relative risk among different ethnic groups, the overlap of confidence intervals across most ethnic groups do not provide strong statistical evidence of effect modification. Thus, pooled sex-specific results are presented in the final column as well in the final columns of Tables 2 and 3. When data for women of different ethnicities were combined (last column of Table 3), a reduced risk for fatal stroke was observed among women who were adherent with the USDA dietary recommendations for vegetable intake, although this result did not reach statistical significance (adherent versus non-adherent: RR = 0.84, 95% CI = 0.68-1.04).

## Discussion

Previous reports have demonstrated that risk for stroke mortality varies among ethnic groups [1], and that dietary patterns also differ by ethnicity [8,9]. To date, there is a lack of research on ethnic specific effects of adherence to the recommended daily servings of fruits and vegetables and risk for fatal stroke. African Americans and Latinos were included in the Continuing Surveys of Food Intakes by Individuals (CSFII) 1994–96, but the samples of these ethnic groups were relatively small [22]. The 3^rd^ National Health and Nutrition Examination Survey (NHANES III) included a large sample of African Americans and Latinos born in Mexico, but the results on adherence to recommendations have not yet been published [23]. Furthermore, no national survey has yet investigated these associations among Asians or Pacific Islanders, who are estimated to comprise 5% of the U.S. population [24]. Neither NHANES nor CSFII sampled populations in Hawaii, a state with a clustering of Japanese Americans [22,23]. Thus, the MEC study provided a unique opportunity to explore the associations between risk for stroke mortality and dietary intake among a very large sample representing these ethnic groups in the United States.

In the current study, statistically significant associations were observed between adherence to dietary recommendations for fruit intake and risk for fatal stroke among Japanese American women. These effects were not observed in Japanese American men, nor any other ethnic-sex group. The apparent stronger association among a specific ethnic group could be attributable to differences in the specific types of fruit and vegetables consumed among the ethnic-sex groups. For example, chili and olives were identified among the top vegetable sources only for Latino-Mexico men and Latino-US men and women, while mangos and papaya are were among the top 10 fruit sources for Japanese Americans and Native Hawaiians only [Sharma, publication under review]. Many fruits are high in vitamin C, which has been shown to lower levels of uric acid, which may in turn decrease blood pressure, and subsequently, risk for stroke [25,26]. Potassium, which plays a role in vasodilation [27], and antioxidants which have been demonstrated to have antiatherosclerotic effects [28], have also been shown to reduce risk for stroke [29,30]. Although analysis of sub-groups was outside the scope of the current study, this factor should be considered in future work.

The current observations also suggest that vegetable intake may be a preventive factor for the risk of fatal stroke in all women, although the results were not statistically significant. Similar results regarding the preventive effects of fruits and vegetables for risk of stroke have been reported previously. A meta-analysis published in 2005 found that the risk of stroke decreased by 5% for each additional serving of fruit and vegetables consumed per day [6]. More recently, the results from a large prospective study found that high intake of raw fruits and vegetables were associated with a preventive effect against stroke, while no associations were observed for processed fruits and vegetables [5]. Unfortunately, information on food preparation was not available for this analysis, but this factor may have accounted for the relatively weak associations observed in this study.

There was also a statistically significant increase in risk for stroke mortality among African American men who had vegetable intake in the second quintile of adherence, compared to the lowest. Given that there was no apparent trend effect, this observation is likely attributable to a chance finding resulting from the number of statistical comparisons.

Although some weak associations were observed among women, the current findings do not provide evidence that associations between fruit and vegetable intake and risk for stroke mortality differ between men and women. However, there did appear to be stronger protective associations between dietary intake and risk for stroke mortality among women, as compared to the results for men. The variation in strength of associations between diet and stroke mortality could be due to differences in physiologic and biochemical characteristics (e.g., percent body fat, serum cholesterol level, blood glucose, hormonal effects in women, or insulin resistance), genetic differences [31], or differences in type and amount of foods consumed and food preparation methods [5] used. Positive or inverse interactions for several nutrients within the diet could also have affected the association between fruit and vegetable intake and risk of death from stroke differently in men and women.

There are some limitations to this study. The accuracy of the dietary data might have been affected by recall bias. It was expected that under-reporting of total energy intake varied among the ethnic groups. Tomoyasu et al. (2000), for example, found that African Americans under-reported energy less frequently than other racial groups [32]. However, the validation study of the QFFQ used in the MEC showed that it captured total nutrient intake relatively well [17,18]. Previous MEC studies have demonstrated that there are similarities in some of the major food choices among these ethnic groups [33-35], thus adaptation to Western diet among the different ethnic groups may also have influenced how these dietary factors impact disease. Addition of data regarding food choices, including food subgroup information may be useful to further elucidate associations between diet and stroke. Many factors can also influence the accuracy of ICD coding for stroke [36], and some research suggests that misclassification of stroke cases may be as high as 20% [37]. As previous studies have found the effects of diet on ischemic versus hemorrhagic stroke to be similar [7,38,39], and considering the potential misclassification related to ICD coding and relatively small number of stroke cases observed in this study, the current analyses did not differentiate outcomes of ischemic versus hemorrhagic stroke. Selection bias may also be a concern due to the large number of exclusions. Nonetheless, large sample sizes were still maintained for the final analyses, thus considerable differences between those excluded and included would be necessary to have impacted results. The initial response rates varied among the ethnic groups, and thus may also have led to selection bias or impacted generalizability, although MEC participants were found to be highly representative of the population in Hawaii and Los Angeles when comparing the cohort distributions across educational levels and marital status with corresponding census data [17]. In addition, dietary patterns may change considerably over a lifetime, and thus the single assessment of dietary intake may not be reflective of long-term dietary exposure. Finally, although the sample size of the MEC study is large enough to support analyses of various outcomes, this study was specifically designed to assess associations between diet and cancer risk, rather than stroke.

Several strengths of this study also warrant mention. It is the first large prospective study to investigate the associations between risk of fatal stroke with adherence to the USDA dietary recommendations for fruit and vegetable intake among these five ethnic groups. The use of a validated QFFQ and a single FCT, which were specifically developed for this multiethnic population, as well as a standard method of food grouping for all ethnic groups based on national recommendations [13], should also facilitate comparisons with other studies. The availability of information on a wide variety of covariates allowed adjustment for possible confounders.

## Conclusions

The results of this study do not provide evidence that dietary intake of fruits and vegetables differentially impacts risk for stroke mortality among different ethnic groups.

Nonetheless, as the percentage of minority ethnic groups in the U.S. increases, additional information on ethnic-specific stroke risk factors are essential. It is important to identify subgroups within the U.S. population at higher risk, as well as to examine the potential impact of cost-effective modifiable risk factors such as diet.

## Competing interests

The authors declare that they have no competing interests.

## Authors’ contributions

The research was designed by LN Kolonel, data were analyzed by S Sharma. S Sharma drafted the manuscript, and M Pakserescht M, JK Cruickshank and DM Green contributed to methodology and critical review of content. All authors read and approved the final manuscript.

## Pre-publication history

The pre-publication history for this paper can be accessed here:

http://www.biomedcentral.com/1471-2377/13/120/prepub
